# Epigenetic Regulation in Heterosis and Environmental Stress: The Challenge of Producing Hybrid Epigenomes to Face Climate Change

**DOI:** 10.3390/epigenomes7030014

**Published:** 2023-07-24

**Authors:** Fátima Duarte-Aké, Rosa Us-Camas, Clelia De-la-Peña

**Affiliations:** 1 Unidad de Biotecnología, Centro de Investigación Científica de Yucatán, Calle 43 No. 130 x 32 y 34. Col. Chuburná de Hidalgo, Mérida 97205, Mexico; 2 Departamento de Estudios de Posgrado e Investigación del Instituto Tecnológico Superior del Calkiní en el Estado de Campeche (ITESCAM), Av. AH Canun S/N San Felipe, Calkiní 24900, Mexico

**Keywords:** DNA methylation, histone posttranslational modifications, sRNAs, heterosis, plant vigor

## Abstract

Epigenetic regulation has the potential to revolutionize plant breeding and improve crop yields by regulating gene expression in plants. DNA methylation and histone modifications are key epigenetic modifications that can impact plant development, stress responses, productivity, and yields. Higher-yielding crops not only generate greater profits for farmers and seed producers, but also require less land, water, fuel, and fertilizer than traditional crops for equivalent yields. The use of heterosis in crops can influence productivity and food quality, but producing hybrids with superior agronomic traits to their parents remains challenging. However, epigenetic markers, such as histone methylation and acetylation, may help select parental and hybrid combinations with better performances than the parental plants. This review assesses the potential applications of epigenetics in crop breeding and improvement, rendering agriculture more efficient, sustainable, and adaptable to changing environmental conditions.

## 1. Introduction

Climate change and the limited availability of arable land present significant challenges to global agriculture [1]. As the world population continues to grow, the demand for food is rising, necessitating agricultural practices that ensure food security. In response to this pressing issue, genetic improvement through hybridization has played a crucial role in increasing agricultural production, starting from the Green Revolution to the present day. Heterosis, achieved by crossing two genetically distant lines in a controlled manner, has been widely implemented in various aspects of genetic improvement to establish desirable traits in agriculturally significant individuals.

The phenomenon of heterosis has been extensively studied and exploited in agriculture, leading to the development of hybrids that exhibit superior phenotypic traits compared to their parent lines. These traits include increased biomass production, enhanced growth rates, higher grain yields, and improved stress resistance [2,3,4]. The basis of heterosis lies in the combination and interaction of favorable alleles from differential parental lines. When two genetically distant lines are crossed, the resulting hybrid inherits a diverse set of genetic material, which can lead to increased genetic variation and novel gene combinations [5,6]. This genetic diversity often results in hybrid plants with improved growth, development, and overall fitness.

Two models have been proposed to explain the generation of hybrids with superior phenotypes. The first model is the dominance model, which suggests that recessive alleles at different loci are complemented in the hybrid. In the extreme form of this model, one parent may possess gene copies that are absent in the other parent, resulting in the hybrid containing more genes than either parent [7]. The second model is known as the overdominance model, which proposes that interactions between different alleles occur in the hybrid, leading to increased vigor [8]. There is also a concept known as pseudo-overdominance, which suggests that complementation occurs for different recessive alleles that are closely linked but located on opposite homologous chromosomes, thereby giving the appearance of overdominance operating [8]. Despite extensive studies on these models, there is still no consensus on how heterosis influences the segregation of outstanding characteristics in the offspring.

The utilization of heterosis in breeding systems has made a significant contribution to the achievement of higher yields in various crop species worldwide, including hybrid rice, maize, canola, sorghum, sunflower, and vegetables [3,9,10]. Continuous advancements in agronomy, such as agricultural machinery and fertilizers, have facilitated heterosis research, while ongoing improvements in breeding systems, such as the development of double-haploid methods, have accelerated the production of inbred lines [11]. The basis of heterosis is a complex interplay of genetic, epigenetic, and physiological factors [2,12].

**Figure 1:**
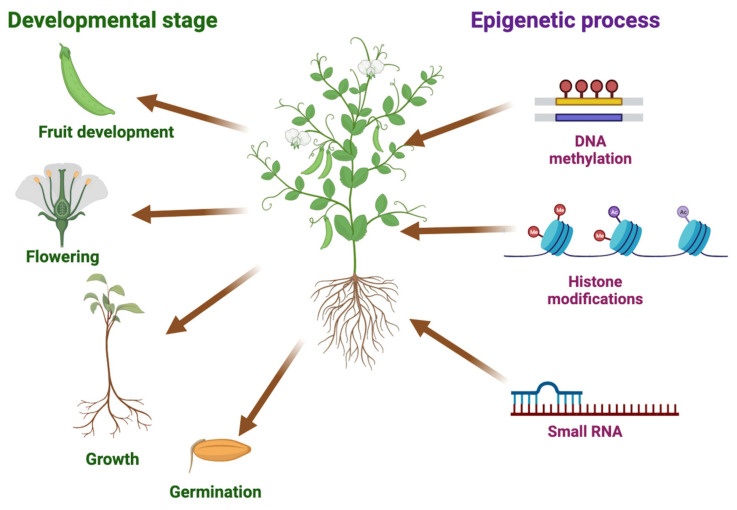
Importance of epigenetic processes to key plant processes. The illustration highlights the significance of epigenetic mechanisms in the regulation of crucial agronomic traits in crops. Different plant processes, such as germination, growth, flowering, and fruit development, are impacted by DNA methylation, histone modifications, and small RNAs in gene expression, which subsequently influences crop productivity, yields, and quality. We used BioRender (BioRender.com) to create this scientific illustration.

Recent studies have highlighted the influence of epigenetic control on heterotic effects in both model and crop plants, expanding our knowledge beyond genetic compatibility. Epigenetic mechanisms, including DNA methylation, histone modifications, and small RNAs, play a crucial role in regulating the expressions of genes associated with important agronomic traits. These epigenetic regulations have been shown to impact essential processes, such as seed germination, plant vigor, growth and development, flowering, fertility, and plant immunity against various pathogens, including bacteria, fungi, and viruses. Additionally, epigenetic regulation plays a critical role in enabling crops to respond to diverse abiotic stresses, such as drought, heat, cold, salinity, and nutrient deficiency [13,14,15,16,17,18,19,20,21,22,23]. Hence, understanding and manipulating epigenetic processes hold great potential for improving crop productivity, yields, and quality, ultimately contributing to sustainable agriculture (Figure 1).

Furthermore, further investigation is required to elucidate the ability of DNA methylation, histone modification, and small RNAs to modulate heterotic effects beyond Mendelian inheritance. The epigenomes of significant crop species can serve as key regulators of agronomical traits, and harnessing this knowledge in crop development can bring significant benefit to farmers and consumers [21]. Additionally, the impact of environmental exposure on plant epigenetic states has been well documented, with epigenetic responses having implications for plant growth, development, and agronomic characteristics [1,24].

## 2. Epigenetic Regulation

### 2.1. DNA Methylation

DNA methylation is a well-studied epigenetic mechanism that regulates gene expression by altering chromatin conformation. When a specific DNA region is enriched with a methylation mark, the chromatin adopts a closed configuration, leading to gene silencing. Conversely, in the absence or reduced presence of methylation, the chromatin assumes an open configuration, promoting gene expression. This mechanism involves the addition of a methyl group to the fifth carbon of cytosine and is catalyzed by enzymes known as methyltransferases (METs). In plants, DNA methylation occurs in three different sequence contexts: CG, CHG, and CHH, where H represents any nucleoside except guanine [25]. Maintenance and de novo DNA methylation take place during DNA replication. CG and CHG methylation patterns are maintained by DNA METHYLTRANSFERASE 1 (MET1) and CHROMOMETHYLASE 3 (CMT3), respectively, while asymmetric CHH methylation is established through de novo methylation catalyzed by CMT3 and DOMAINS REARRANGED METHYLTRANSFERASE 2 (DRM2) [26,27]. Additionally, DRM3 plays a crucial role in initiating de novo cytosine methylation in all sequence contexts through a process called RNA-directed DNA methylation (RdDM) [28]. In the RdDM pathway, a complementary sequence within a heterochromatic region is transcribed by POL IV, leading to the synthesis of small RNAs (ssRNAs). These ssRNAs are then converted into double-stranded RNAs (dsRNAs) through the action of RNA-directed RNA polymerase 2 (RDR2). The dsRNAs are subsequently sliced into 24-nucleotide fragments, and one strand from each 24-nucleotide double-stranded small RNA is loaded into ARGONAUTE (AGO), forming an RNA–protein complex that recognizes and binds to complementary target sequences. This interaction recruits DRM3, which methylates the neighboring DNA [29]. The CHH context is dependent on 24-nucleotide small interfering (si)RNAs to guide the methyltransferases, or on a second pathway involving DEFICIENT IN DNA METHYLATION 1 (DDMI) and CMT2 [30].

### 2.2. Histone Modifications

Eukaryotic DNA is packaged into chromatin, which is composed of nucleosomes consisting of histones H2A, H2B, H3, and H4 [13,31]. The *N*-terminal tails of histones are subject to different posttranslational modifications, including acetylation, methylation, ubiquitination, phosphorylation, biotinylation [32], adenosine diphosphate (ADP)-ribosylation [33], crotonylation [34], and sumoylation [35], among others [13,31,36,37], which regulate the chromatin structure and accessibility to DNA. In plants, histone acetylation usually occurs at lysine residues of histones H3 and H4 and is associated with transcriptional gene activation [16,18]. Histone methylation, such as H3K9me2 and H3K27m3, is linked to gene repression [13,38], while the H3K36me2 and H3K36me3 marks are associated with gene transcriptional activation [13,39,40]. Overall, histone posttranslational modifications contribute to the establishment of a histone code that regulates gene expression and the chromatin structure [32].

### 2.3. Small RNAs

Gene expression and epigenetic control are regulated by small RNAs, including microRNAs (miRNAs), small interfering RNAs (siRNAs), and trans-acting small interfering RNAs (ta-siRNAs). MiRNAs are short regulatory RNAs, approximately 19–24 nucleotides in length, that negatively regulate gene expression. MiRNAs are synthesized by DNA-dependent RNA Pol II from MIR genes and are derived from a hairpin or stem–loop precursor [41,42,43]. The primary miRNA (pri-miRNA) is first cleaved by the RNase III DICER-Like1 (DCL1) to create the intermediate precursor pre-miRNA [44]. DCL1 then cleaves the pre-miRNA to form the mature miRNA duplex along with the dsRNA-binding protein HYPONASTIC LEAVES1 (HYL1) in the nucleus [41]. The nuclear methyltransferase HUA ENHENCER1 (HEN1) attaches a methyl group to the 2′ OH of the mature duplex miRNA’s 3′ last nucleotide. The Arabidopsis EXPORTIN5 ortholog HASTY transports the miRNA from the nucleus to the cytoplasm and the methyl groups are removed in the cytoplasm. A helicase unwinds the double-stranded mature miRNA to produce a single-stranded mature miRNA that is recognized by ARGONAUTE 1 (AGO1) [41]. AGO1 recruits the entire RNA-induced silencing complex (RISC) that recognizes the mRNA targets that the mature miRNA regulates [41,45]. AGO1 works with *AMP1* (*ALTERED MERISTEM PROGRAM1*) to suppress the translation of target mRNAs in the endoplasmic reticulum [46,47]. The biogenesis of miRNAs can produce two types of miRNAs: those that perfectly complement their mRNA targets and those that have mismatches with their targets. MiRNAs with perfect matches to their target mRNAs induce mRNA cleavage, while miRNAs containing mismatches suppress translation by binding stably to the mRNA targets [42,43,48].

## 3. Epigenetic Mechanisms and Heterosis

Despite the extensive use of hybrids in agriculture, the mechanisms underlying heterosis are not fully understood. One proposal is that dominance and/or overdominance between alleles from the two parental lines result in the heterozygote’s advantage [8]. However, recent genetic and molecular evidence suggests that epigenetic mechanisms may also play a role in heterosis. For example, experiments on *Arabidopsis thaliana* have shown that even when the two parental lines have a close genetic relationship, with little genetic distance between them [49,50], the hybrids that emerge demonstrate pronounced heterosis, characterized by enhanced levels of both vegetative biomass and seed yield [51]. This is because the two parental lines have extensive epigenetic differences [52,53,54], resulting in an altered gene expression pattern in the hybrid [55].

The occurrence of DNA methylation changes between the *C24* accession and *Ler* accession (*Landsberg erecta*) is predominantly context-specific and is observed at loci where the two parental epialleles exhibit distinct methylation frequencies. In some cases, a hypermethylated segment in *C24* can be allelic to a hypomethylated segment in the *Ler* parent. Consequently, the methylation patterns of the *C24* parent can be transferred to the *Ler* allele within the hybrid nucleus, resulting in methylation events referred to as Trans Chromosomal Methylation (TCM), or demethylation events known as Trans Chromosomal Demethylation (TCdM) [52].

While a significant proportion of loci meeting the criteria for differential methylation between the two parents do not exhibit TCM or TCdM events in the hybrid, it is noteworthy that certain events can indeed lead to changes in gene expression [52,54]. Although most of these events may not directly impact gene expression in the hybrid [56], even minor alterations in key regulatory components have the potential to trigger a cascading effect on transcriptome patterns and gene regulation. Supporting this notion, the hybrid transcriptome shares similarities to the transcriptomes of mutants with altered methylation patterns [54]. To comprehensively understand the extent to which epigenetic mechanisms contribute to the heterotic phenotype and explore their potential application in agriculture, further research is warranted. Investigating the role of epigenetics in heterosis can provide valuable insights into improving crop productivity and developing sustainable agricultural practices.

Recent research has shed light on the crucial role of histone modifications in heterosis across various plant species (Table 1). A comprehensive analysis of four key histone modifications (H3K4me3, H3K9ac, H3K27me3, and H3K9me2) in *Ler/C24* hybrids and their parental lines indicates that these modifications are involved in regulating the expressions of specific genes in hybrids. Notably, in Arabidopsis, the reduced expression of *FLOWERING LOCUS C* (*FLC*), a negative regulator of the flowering time, was associated with decreased levels of H3K27me3, resulting in delayed flowering. This link between histone modifications and the flowering time provides insights into the potential influence of heterotic traits [57] (Figure 1).

Moreover, in *Arabidopsis*, the circadian clock is regulated by transcription factors such as the *LATE ELONGATED HYPOCOTYL* (*LHY*), *CIRCADIAN CLOCK ASSOCIATED 1 (CCA1)* genes, and regulators such as *TIMING OF CAB EXPRESSION 1* (*TOC1*) and *GIGANTEA* (*GI*). *CCA1* and *LHY* negatively regulate the expressions of *TOC1* and *GI*. During daylight hours, in vigorous plants, *CCA1* and *LHY* were repressed while *TOC1* and *GI* were upregulated. An analysis of the upstream region (approximately 250 bp) of *CCA1* and *LHY* showed a two-fold reduction in the transcriptional activation marks H3K9ac and H3K4me2 that was correlated with the repression of *CCA1* and *LHY*. In contrast, *TOC1* and *GI* showed an increase in H3K9ac and H3K4me2 that correlated with the increase in their expressions. The analysis of genes downstream of *CCA1* and *LHY* showed that genes related to chlorophyll biosynthesis (*PORA* and *PORB*) and starch metabolism (*AMY3*, *BAM1*, *BAM2*, *DPE1*, *DPE2*, *GTR*, *GWD1*, *GWD3*, *ISA1*, *ISA2*, *ISA3*, *LDA*, *MEX1*, *PHS1*, and *PHS2*) increased their expression levels, resulting in more chlorophyll, starch, and sugars in the F1 hybrid than in the parents [58].

In hybrid rice lines that showed vigorous growth, the H3K4me3 mark correlated positively with differentially expressed genes, but a weak correlation was observed between H3K27me3 and the differential gene expression compared to parental inbreeds [59]. Furthermore, parental epialleles were faithfully transmitted to the F1 hybrid lines (Guangluai (GL) × 93-11 and GL × Teqing (TQ)) of rice, contributing strongly to allele-specific histone modifications (ASHMs) in the F1 hybrids. ASHM-H3K36me3 contributes to and regulates allele-specific gene expression in F1 hybrids. The expressed monoallelic genes contained the H3K36me3 modification, and a strong correlation between allele-specific gene expression was observed with H3K36me3 but not with H3K27me3 [60].

In maize embryos and endosperm, the accumulation of the histone 2A (H2A) variant, HTA112, was found to differ between hybrid genotypes and inbred parents, suggesting a potential epigenetic association with heterosis in maize [61]. These findings highlight the crucial role of histone modifications in regulating gene expression and promoting heterosis in plants (Table 1).

Short RNAs, such as miRNAs and siRNAs, have been implicated in contributing to hybrid vigor [54,62]. Non-additive expression patterns have been observed in most miRNAs found in hybrids, indicating their contribution to the robustness and adaptability of hybrids. In some cases, changes in miRNA expression can lead to increased vigor, as demonstrated in the F1 hybrid of *Brassica napus* and Chinese cabbage [63,64] (Table 1). In hybrid maize with a deficiency in Mediator of Paramutation1 (MOP1), a protein essential for the synthesis of 24-nucleotide (24 nt) siRNAs [65], the ability to exhibit heterosis remained unaffected. This suggests that reduced levels of 24 nt siRNAs and alterations in methylation may contribute to the heightened vigor observed in hybrids [62].

The involvement of 24 nt siRNAs in hybrid vigor is multifaceted, and their role is not solely determined by their abundance. This is exemplified by the Arabidopsis *hen1* mutants, which exhibit a global decrease in 24 nt siRNAs and display a reduced size. In hybrids involving *hen1* mutants, the compromised vegetative vigor suggests that a decrease in 24 nt siRNAs alone does not confer hybrid vigor. The dwarfed phenotype observed in *hen1* mutants implies that developmental defects resulting from reduced miRNA and 24 nt siRNA levels in the genome impede growth, potentially masking the manifestation of heterosis [54,66].

Studies have demonstrated that in hybrids, there is a downregulation of 24 nt siRNAs specifically in genome regions where the parental lines differ in siRNA levels [55,59,62,67]. These differences in siRNA regulation may be limited to specific tissue types, as observed in maize, where the decrease in siRNAs is restricted to the differentiated developing ear and is not observed in the meristematic shoot apex [62]. Notably, the decrease in 24 nt siRNAs is not observed in other size types of small RNA. In hybrid systems involving closely related parental lines, the miRNA expression tends to exhibit additive patterns [55,62,67]. However, hybrids derived from more divergent parental lines display non-additive expressions of several miRNAs. These non-additive expressions can potentially influence the gene expression and phenotypic development of F1 hybrids [68,69].

Distinct patterns of miRNA expression have been revealed in hybrid crops compared to their parental lines, indicating the involvement of miRNAs in heterosis [2]. Recent research on *B. napus* F1 hybrids demonstrated higher expression levels of major miRNA clusters in hybrids than those in their parents, highlighting their role in plant growth and vigor [63]. In Chinese cabbage, Li et al. [64] identified heterosis-regulating miRNAs and genes, along with their target transcripts, using an analysis that enables the identification and characterization of specific target transcripts that are degraded by microRNAs (miRNAs). Among the upregulated genes found in the F1 hybrid transcriptome was *LIGHT-HARVESTING COMPLEX OF PHOTOSYSTEM II* (*LHC*), associated with an enhanced photosynthesis capacity through larger cells and increased granum thylakoids [70,71,72]. Notably, the repression of bra-miR5722, targeting *BrLHCB1.2*, in the F1 hybrid indicates miRNA-mediated regulation contributing to the improved photosynthesis capacity associated with heterosis. MiRNAs with implications for plant growth and vigor have been differentially regulated in hybrids across various species, such as Arabidopsis, wheat, and *B. napus* [63,68,73].

**Table 1:** Epigenetic processes in heterosis in different plant species.

Epigenetic Process	Plant Species	Function	Reference
**DNA methylation**	*Arabidopsis thaliana*	Alters DNA methylation patterns, specifically ^m^CG and ^m^CHH islands, which are associated with reduced 24 nt siRNA levels and contribute to heterosis in terms of increased biomass and seed yield.	[52]
		Enhances DNA methylation in specific genes, such as *CIRCADIAN CLOCK ASSOCIATED1* and *LATE ELONGATED HYPOCOTYL*, regulated by the RNA-directed DNA methylation (RdDM) pathway, promoting growth vigor in hybrids.	[54]
	*Oryza sativa*	Induces transgenerational epimutations across genetically identical chromosomes and generations, contributing to heterosis.	[56]
**Histone modification**	*Arabidopsis thaliana*	Represses the transcription-factor genes *LATE ELONGATED HYPOCOTYL* (*LHY)*, *CIRCADIAN CLOCK ASSOCIATED 1* (*CCA1*) through the reduction in H3K9ac and H3K4me2 marks, leading to enhanced expressions of genes involved in chlorophyll biosynthesis and starch metabolism, thereby promoting growth vigor.	[58]
		Delays flowering by allowing the expression of *FLC* (*FLOWERING LOCUS C*), controlled by reduced levels of H3K27me3, contributing to heterosis in terms of flowering traits.	[57]
	*Oryza sativa*	Shows a positive correlation between hybrid vigor and the H3K4me3 mark, impacting gene expression, while exhibiting minimal correlation with the H3K27me3 mark, contributing to growth vigor.	[59]
		In F1 hybrid, allele-specific histone modifications (ASHMs) like H3K36me3 regulate allele-specific gene (*ASE*) expression. The epialleles associated with ASHMs play a significant role.	[60]
	*Zea mays*	Displays differential expression of *HTA112*, a histone 2A (H2A) variant, in hybrid genotypes compared to inbred parents, influencing early seed germination processes.	[61]
**Small RNA**	*Arabidopsis thaliana*	Correlates the reduction in 24 nt siRNAs with changes in DNA methylation and gene expression, contributing to hybrid vigor in terms of enhanced plant vigor.	[67]
	*Brassica napus*	Increases the expression levels of small interfering RNA (siRNA) clusters in hybrids, leading to changes in methylation levels and reduced expressions of transposable elements (TEs), contributing to heterosis in early flower development.	[63]
	*Brassica rapa* L. spp. *pekinensis*	Reduce expression levels of most miRNA clusters, influencing the target genes involved in photosynthesis and chlorophyll synthesis, resulting in increased photosynthesis capacity and improved biomass, contributing to heterosis.	[64]
	*Zea mays*	Maintains hybrid vigor when 24 nt siRNAs are globally reduced through the mutation of *mop1* (*modifier of paramutation1*), an RNA-dependent RNA polymerase 2, ensuring the sustained expressions of advantageous traits related to plant vigor.	[62]

## 4. Factors Affecting Epigenetic Mechanisms and, Therefore, Productivity

**Figure 2:**
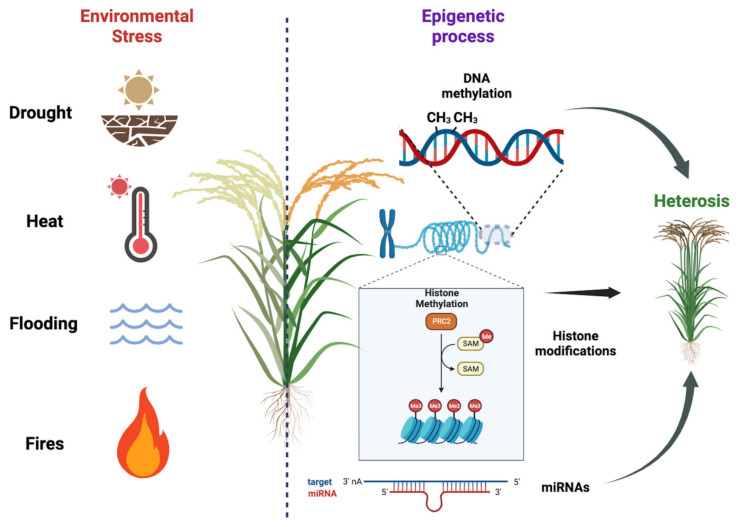
Plant responses to environmental stresses and the importance of epigenetic regulation on hybrid vigor. Plants face various environmental stresses, impacting growth and yields. Developing stress-resistant cultivars is crucial for agriculture. Understanding plant stress responses and epigenetic regulation, including DNA methylation, histone modification, and miRNA regulation, helps identify targets for crop enhancement. We used BioRender (BioRender.com) to create this scientific illustration.

Plants are constantly exposed to various environmental stresses, such as nutrient deficiency, drought, heat, salinity, and soil contamination with heavy metals. These stressors can have detrimental effects on plant growth, biomass production, and overall yields (Figure 2). In order to mitigate losses in agriculture, it is imperative to develop stress-resistant cultivars that can better withstand these challenging conditions. A key aspect in achieving this goal is gaining a deeper understanding of plant stress responses and their regulation, specifically focusing on the chromatin states and histone modification that govern gene expression. By unraveling these epigenetic mechanisms, researchers can uncover novel targets for crop enhancement, leading to the creation of more productive and resilient plants capable of adapting to changing environmental conditions. This research is of the utmost importance for improving agronomic traits and enhancing productivity, thereby ensuring food security in the face of evolving climate change and other environmental pressures [1,74].

### 4.1. Heat Stress

Temperature is a crucial environmental factor affecting plant growth, biomass, and yields. Temperature changes, both heat and cold, pose a significant challenge to agriculture. Heat stress, in particular, can lead to morphological, physiological, and biochemical changes in plants, including growth retardation, leaf etiolation, and even death [75]. Heat stress induces signaling cascades and triggers the expressions of specific genes [76] and heat-shock proteins (HSPs) [77]. Studies have shown that different plant genotypes exhibit varying degrees of heat tolerance.

The responses of plants to temperature stress involve epigenetic mechanisms, specifically histone posttranscriptional modifications [23,78,79]. To investigate the impact of heat stress on methylation patterns, researchers have examined methylation levels and changes in cytosine methylation patterns in seedlings of heat-sensitive and heat-tolerant genotypes. The findings revealed that the methylation levels differed between the heat-tolerant and heat-sensitive phenotypes under normal conditions [80]. Upon exposure to heat treatment, methylation increased to a greater extent in the heat-sensitive genotype compared to the heat-tolerant genotype. Interestingly, DNA demethylation events were more prevalent in the heat-tolerant genotype, whereas DNA methylation occurred more frequently in the heat-sensitive genotype. This suggests that changes in DNA methylation patterns are associated with the heat-stress response and adaption in *B. napus* L. [81] (Table 2). Intriguingly, through the use of an MSAP assay, a polymorphic demethylated fragment known as M7 (digested with *Eco*RI/*Msp*I) was identified that was found to be linked to a calcium-transporting ATPase gene. This gene plays a crucial role in facilitating the direct transport of calcium ions [82]. The primary calcium-transporting ATPase present in the plasma membrane and endoplasmic reticulum of plant cells utilizes ATP hydrolysis to transport calcium ions. Thus, the alteration of the Ca^2+^ concentration in the cytoplasm due to stress could serve as a primary transduction mechanism, influencing gene expression and biochemical events to enable plant cells to adapt to environmental stresses, including heat stress [83].

The role of histone acetylation, mediated by histone acetyltransferases (HATs) and histone deacetylases (HDACs), has been highlighted in the response to heat stress [15,18] (Table 2). Heat stress triggers thermomorphogenesis in *Arabidopsis,* characterized by elongated growth and early flowering, enhancing the cooling capacity of the plant [15,84]. HDACs, such as HDA9, play a crucial role in thermomorphogenesis by promoting the expressions of genes involved in this response. For instance, HDA9 interacts with PRW (POWERDRESS) to increase the deacetylation of H3K9 at specific gene loci, such as *PHYTOCHROME INTERACTING FACTOR4 (PIF4)* and *YUCCA8 (YUC8)*, which are essential for thermomorphogenesis [85] (Table 2). HDA9 activity is also required for *YUC8* expression via the promotion of the eviction of the histone variant H2A.Z from *YUC8* nucleosomes, leading to histone deacetylation at the transcriptional start site and gene body of *YUC8* and allowing its transcriptional activation by PIF4 [86]. These findings suggest that histone acetylation and deacetylation could be a valuable strategy for enhancing crop yields under heat-stress conditions, thereby potentially impacting heterosis.

In *Arabidopsis,* the activity of HDA15 has been shown to act as a repressor of the response induced by warm temperatures [87], while HDA9 and HDA19 appear to participate indirectly in the response to the same stimulus [88]. At 27 °C, *hda15* mutant seedlings showed elongated hypocotyls compared to Col-0 plants, while the hypocotyls were shorter in *hda9* and *hda19* mutant seedlings. Furthermore, warm-temperature marker genes, such as *HSP20*, *IAA3*, *IAA19*, *IAA29*, *YUC8*, *SAUR28*, and *TCH3*, were upregulated in the *hda15* mutant compared to the *hda9* and *hda19* mutants and Col-0 plants. In addition, *HSP20*, *IAA19*, and *IAA29* genes showed increased levels of H3K14ac in their promoter and 5′ regions. At 20 °C, the *hda15* mutant also showed the upregulation of warm-temperature marker genes, such as *YUCCA8, IAA19*, *IAA29*, *TCH3, ATHB2*, and *XTR7*. These results suggest that HDA15 can repress warm-temperature marker genes during normal growth and dissociate from its targets to induce their expressions under elevated-temperature stimuli [88].

### 4.2. Drought Stress

Water deficiency is a major challenge in agriculture, and plants have been found to respond to this stress through epigenetic modifications, including histone acetylation [16,89]. The dynamic activity of HATs/HDAs regulates the response to drought stress in important crops such as rice, wheat, and cotton [81,90,91]. In *Arabidopsis*, H3K9ac has been shown to positively regulate the expressions of drought-response genes [92]. The dynamic activity of HATs and HDAs also regulates the ABA biosynthesis pathway, which is the most important signaling pathway for drought stress in plants and is found in various plant species [16,18].

Epigenetic associations with heterosis in response to drought stress have also been observed [10,93]. A study conducted on poplar (*Populus euramericana*) examined six hybrid genotypes (*P. deltoides* × *P. nigra*) subjected to water-deficit conditions. The results revealed a correlation between the morphological traits related to productivity and epigenetic modifiers under drought stress. In the hybrid genotype *Populus deltoides* × *P. nigra*, the hypomethylation of DNA was found to be associated with drought stress, while there was a significant increase in histone acetylation, indicating rapid gene expression potentially linked to heat-shock proteins (HSPs) **[93]** (Table 2). These findings highlight the potential role of epigenetic mechanisms in mediating heterosis and enhancing drought-tolerance traits in plants.

Various studies have shown a positive correlation between increased *HAT* expression and drought tolerance in plants [16,18]. In *Brassica rapa,* the expressions of nine *HAT* genes, including *BraHAC1*, *BraHAC2,* and *BraHAC3*, increased significantly after two and/or four days of drought treatment [94]. Similarly, in *Brachypodium distachyon* and *Oryza sativa,* the expressions of five HATs (*BdHAG1*, *BdHAG3*, *BdHAC1*, *BdHAC4*, and *BdHAF1)* and nine *HATs* (*OsHAG702//703*, *OsHAD704/705/706/711/712/713*, and *OsHAM701*), respectively, were induced after drought treatment [90,95] (Table 2). Analysis of the promoter region of some of these *HAT* genes, such as *OsHAG702*, *OsHDA705/706/713,* and *OsSRT702,* showed the existence of drought-responsive elements, like the MBS *cis-*element (MYB-binding site involved in drought inducibility), indicating the participation of specific transcription factors for gene activation [90]. In wheat, the genes *TaHAG2*, *TaHAG3*, and *TaHAC2*, and particularly *TaHAG2*, showed significantly higher expressions in the drought-resistant variety BL207 compared to its less-resistant parents, BN64 and ZM16. This indicates the potential involvement of these genes in the drought response of wheat [91]. In *Arabidopsis*, drought stress triggered an increase in the H3K9ac levels within the promoter regions of 14 drought-response genes, suggesting a crucial role for H3K9ac in the transcriptional activation of these genes under water-deficit conditions [92]. This mechanism suggests the formation of tertiary protein complexes that enhance gene expression [96].

HDAs generally appear to negatively regulate the expressions of drought-responsive genes. For instance, the HDA9 mutation in Arabidopsis resulted in the upregulation of 47 water-deprivation-response genes and the downregulation of 13 genes compared to wild-type plants. The promoter region of 14 randomly selected upregulated genes in the *hda9* mutant showed increased levels of H3K9ac (>2-fold), indicating that the increased expressions of these genes are due to a decrease in deacetylase activity [92]. Similarly, plants that silenced *AtHDA6* and *AtHDA19* exhibited a hypersensitive phenotype to ABA, resulting in the decreased expressions of ABA-responsive genes (*KAT1*, *KAT2*, *ABI1*, *ABI2*, *RD29A, RD29B*, and *DREB2A*) when treated with ABA [87].

AtHD2C has been implicated in the response to ABA. Transgenic plants overexpressing *AtHD2C* exhibited insensitivity to ABA and demonstrated enhanced drought tolerance compared to wild-type plants. Furthermore, the expression of *AtHD2C* was repressed by ABA [97], and AtHD2C can physically interact with HDA6 and function in association to regulate the expressions of ABA-responsive genes [98]. Recent studies indicate that AtHDA15, through the transcription factor MYB96, can regulate gene responses mediated by ABA signaling [99,100]. The biochemical and molecular mechanisms by which HDA6, HDA9, and HDA15 act to regulate responsive genes for ABA signaling have been described in detail in previous studies [16,99,101,102].

In soybean (*Glycine max*), the expressions of the nine *GmHDACs* (*GmHDA6*, *GmHDA8*, *GmHDA13*, *GmHDA14*, *GmHDA16*, *GmSRT2*, *GmSRT4*, *GmHDT2,* and *GmHDT4*) were found to decrease after drought treatment [103]. Similarly, in rice, the expression of *OsHDA703/710* was significantly decreased after drought treatment [90]. In wheat, the drought-resistant variety BL207 showed a downregulation of the expressions of *TaHDA2*, *TaHDA18*, and *TaHDT2* [91]. However, in some cases, an increase in *HDAC* expression may occur, potentially inhibiting the function of the transcriptional repressors of drought-stress-response genes. For example, in rice, increases in the expressions of *OsHAG702/703*, *OsHAM701*, *OsHDA704/705/706/711/712/713*, *OsHDT701*, and *OsSRT702* were observed after drought treatment [90], and in *Hibiscus cannabinus* L., five *HcHDA* genes (*HcHDA2*, *HcHDA6*, *HcHDA9*, *HcHDA19*, and *HcSRT2*) were strongly expressed under PEG treatment [104].

The use of epigenetic mechanisms offers promising strategies for enhancing drought tolerance in plants. Modulating the expression or repression of *HDAC* has shown significant impacts on drought tolerance in different plant species. For instance, in tobacco, introducing the histone deacetylase 84 KHDA903 from poplar (*Populus alba* × *Populus glandulosa*) resulted in the overexpressions of drought-responsive genes (*NtDREB4*, *NtDREB3,* and *NtLEA*), leading to improved drought tolerance [105]. In cotton, overexpressing the histone deacetylase *GhHDT4D*, a member of the HD2 subfamily, enhanced drought tolerance by reducing the H3K9ac levels in the promoter region of *GhWRKY33*, a negative regulator of cotton’s response to drought, and suppressing its expression [81]. In *Arabidopsis*, *AtHD2C* and *HDA6* were found to decrease the expressions of ABA-responsive genes by reducing histone H3K9/K14 acetylation and increasing H3K9me2 [98]. Conversely, H3K4me3 appears to play an important role in the response to drought stress in *Arabidopsis*, as the 5′ ends of most ABA and dehydration-inducible genes exhibited broader H3K4me3 distribution profiles [106]. These findings suggest that alterations in HDAC expression and histone modifications are involved in the plant response to drought stress and hold potential for early stress detection. Additionally, manipulating the transcriptional activation or repression of *HDAC* can offer promising avenues for improving drought tolerance across different plant species.

**Table 2:** Plant responses to stress and epigenetic processes in different plant species.

Plant Response	Epigenetic Process	Plant Species	Function	Reference
**Heat stress**	Histone modification	*Arabidopsis thaliana*	HDA9 interacts with the PWR protein and increases H3K9 deacetylation at the +1 nucleosomes of *PHYTOCHROME INTERACTING FACTOR 4* (*PIF4*) and *YUCCA8* (*YUC8*), essential genes regulating thermomorphogenesis.	[85]
			HDA9 promotes the eviction of the histone variant H2A.Z from the *YUC8* nucleosome and enables its transcriptional activation by PIF4, mediating the thermomorphogenic response.	[86]
			HDA15 acts as a repressor of warm-temperature marker genes (*YUCCA8*, *IAA19*, *IAA29*, *TCH3*, *ATHB2*, and *XTR7*) under normal conditions but dissociates from its targets under elevated-temperature stimuli, inducing their expressions.	[88]
	DNA methylation	*Brassica napus*	Exhibits more DNA demethylation events in heat-tolerant genotypes, which are associated with heat-stress response and adaptation.	[80]
**Drought stress**	DNA methylation/histone modification	*Populus deltoides × P. nigra*	Shows genotypic variation in DNA hypomethylation that correlates with morphological traits related to productivity under drought stress. Histone acetylation induces rapid gene expression associated with heat-shock proteins (HSPs) under drought-stress conditions.	[93]
	Histone modification	*Arabidopsis thaliana*	HDA9 negatively regulates plant sensitivity to drought stresses through increased H3K9ac levels in the promoter region of 14 drought-response genes under water-deficit conditions.	[92]
			AtHD2C physically interacts with HDA6 and regulates the expressions of ABA-responsive genes in association.	[97,98]
		*Brachypodium distachyon*	Exhibits increased expressions of five *HAT* genes (*BdHAG1*, *BdHAG3*, *BdHAC1*, *BdHAC4, BdHAF1)* under drought treatment, playing a role in drought-stress response and adaptation.	[95]
		*Brassica rapa*	Demonstrates a significant increase in the expressions of nine *HAT* genes (*BraHAC1*, *BraHAC2*, *BraHAC3*, *BraHAC4*, *BraHAC7*, *BraHAG2*, *BraHAG5*, *BraHAG7*, and *BraHAF1*) after drought treatment, contributing to drought-stress response and adaptation.	[94]
		*Gossypium hirsutum*	Enhanced drought tolerance by reducing H3K9ac levels in the promoter region of *GhWRKY33*, a negative regulator of drought response, through the action of GhHDT4D, a member of the histone deacetylase HD2 subfamily.	[81]
		*Dendrobium officinale*	Induces the expressions of *DoHDA10* and *DoHDT4* genes in roots, stems, and leaves under drought-stress conditions.	[107]
		*Oryza sativa*	Triggers the expressions of nine *HAT* (*OsHAG702//703*, *OsHAD704/705/706/711/712/713*, and *OsHAM701*) genes under drought conditions. Some *HAT* genes contain drought-sensitive elements, such as the MBS cis element, in their promoter regions.	[90]
		*Triticum aestivum*	Demonstrates the downregulation of five *HDA* genes and a significant increase in *TaHAC2* expression in the drought-resistant variety BL207 under drought-stress conditions.	[91]

## 5. Conclusions

Investigations have revealed the significant role of histone acetylation and methylation in regulating various plant development processes, as well as responses to biotic and abiotic stresses and adaptation to changing environmental conditions. These epigenetic modifications have a profound impact on agronomic traits and plant productivity [1,18,74]. Key developmental stages, such as seed germination, vegetative growth, blooming, fruit development, and responses to stressors, are all influenced by histone acetylation and methylation [1,18,74].

Understanding the underlying mechanisms of epigenetics in plant responses holds tremendous potential to revolutionize crop breeding and improve overall plant productivity, particularly in the face of environmental changes. Increased crop yields not only benefit farmers and seed producers financially but also have positive environmental implications by reducing land, water, fuel, and fertilizer requirements [108]. The complex phenomenon of heterosis can be better understood through the study of epigenetic mechanisms, particularly histone methylation and acetylation, which may pave the way for the development of novel hybrid plant varieties with superior agronomic traits [17]. Leveraging epigenetic markers and artificial epigenome editing techniques could enhance the selection of new crop variants and streamline crop-breeding programs [21,74,109]. Moreover, insights gained from investigating histone modifications and their regulation of plant responses to biotic stressors could aid in the development of stress-resistant crop varieties with enhanced adaptability to changing environmental conditions, ultimately boosting agricultural production and food quality. Thus, the applications of epigenetics in agriculture are vast, and further research in this field holds great promise for the benefit of both farmers and the environment.

Temperature fluctuations and water limitations are two major abiotic factors that significantly influence plant growth, development, productivity, and food quality [18,110,111]. Emerging evidence suggests that histone acetylation, particularly mediated by HATs and HDAs, plays a crucial role in the responses of plants to environmental stress conditions caused by temperature variations and drought. *HDAs* regulate temperature-induced morphological changes and the plant heat response [15]. In economically important crops, such as rice, wheat, and cotton, different members of the *HAT*/*HDA* family modulate the response to drought stress by influencing the expressions of drought-responsive genes [16,18]. Expanding our knowledge of histone acetylation and its regulatory mechanisms enables the targeted manipulation of specific HATs/HDAs through chemical or molecular approaches, facilitating the generation of new crop varieties adapted to water-limited and arid regions prone to temperature fluctuations. This knowledge can aid traditional breeding methods and contribute to the development of effective crop improvement strategies in breeding programs.

## Data Availability

No new data were created or analyzed in this study. Data sharing is not applicable to this article.
